# Design of custom-made navigational template of femoral head and pilot research in total hip resurfacing arthroplasty

**DOI:** 10.1186/s12893-020-00807-7

**Published:** 2020-06-30

**Authors:** Jinlong Liang, Yonghui Zhao, Xinjian Gao, Xuewei Fang, Yongqing Xu, Sheng Lu

**Affiliations:** 1 Department of Orthopedics, 920 Hospital of Joint Logistic Support Force, Kunming, 650032 China; 2 Xi’an Jiaotong University, Xian, 710049 China; 3 Department of orthopedics, the first people’s hospital of yunnan province; Yunnan Provincial Key laboratory of digital orthopedics, Kunming, 650032 China

**Keywords:** Custom-made navigational template, Total hip resurfacing Arthroplasty, Computer assisted surgery, Rapid prototyping, Anatomical axis of the femoral neck

## Abstract

**Background:**

To develop a novel custom-made navigational template for accurate prosthesis implantation in total hip resurfacing arthroplasty (THRA) by computer-aided technology.

**Methods:**

The template was produced based on data preoperatively acquired from computed tomography (CT) scan. The position of the drill guide was obtained according to the anatomical axis of the femoral neck which was defined by the point of the femoral head center and another point of the femoral neck center. The final direction of the drill guide was confirmed by a valgus angle. The surface of the template was constructed based on the inverse of the femoral neck surface. Then the template was made of acrylate resin by using rapid prototyping (RP) technique. Finally, all the templates were verified in 17 cadavers arranged for THRA and postoperative medical images were employed to evaluate the accuracy and validity of the template.

**Results:**

The templates had achieved a high fitting with the femoral neck surface, and there were no guide failures. Postoperative evaluation revealed that the Kirschner-wires pass through the center of the femoral head and femoral neck, presenting a relative expected and acceptable valgus angle to the central axis of the femoral neck. The lateral offset showed the relative valgus angle achieved as expected, the horizontal offset showed that no obvious antero-posterior deviation occured. The comparison between the preoperative Neck-shaft angle (NSA) and the postoperative Stem-shaft angle (SSA) showed there is no significant difference(*P* > 0.05).

**Conclusion:**

The novel custom-made navigational template of femoral head can effectively assist surgeons for accurately implanting the femoral head components to the desired position in THRA.

## Background

Total hip arthroplasty (THA) requires remove the femoral head and is the most frequently used among patients affected by hip diseases [1], it is considered to be a well-accepted treatment that provides pain relief and functional restoration to the most patients [2, 3], but the long-term survivial of prostheses and the occurrence of postoperative complications remain challenges to the surgeons [4]. Therefore, total hip resurfacing arthroplasty (THRA) has been developed for younger and more active patients, especially for proximal femoral bone stock preservation [5]. It has the advantage of preserving the bone stock and restoration of the native anatomy [6]. However, several studies have shown that early postoperative femoral neck fracture occurred and was related to improper surgical operations and femoral prosthesis implantations which influenced the biomechanic characteristic of the hip [7, 8]. Thus, the location of the femoral prosthesis is vital for implant survival. However, only a few methods have been developed for guiding the orientation of the femoral prosthesis in THRA [9].

In recent years, Rapid prototyping, or 3D printing template has been widely used to assist surgery. It provides the optimal functional and anatomic outcomes, patient satisfaction, and precise translation of the preoperative planning [10]. Some authors have applied it to places cervical pedicle screws [11], or to guide the localization of lung nodules in percutaneous procedures [12]. The template eliminates the need for complex equipment and time consuming procedures intraoperatively, and makes the precise, safe, and fast implantation of the prostheses possible [13]. The custom-made template can be user friendly as well as successfully applied with accurate guide according to the preoperative planning. It can transfer a preoperative plan into surgery with improved accuracy of positioning of the orthopedic plant component [14].

The demand for accurate joint replacement has been continuously increasing, particularly for prosthesis locating. Considering the different anatomical types of the hip joint, individualized treatments can optimally adapt and accurately treat pathological joint disease. The aim of this study was to develop a novel patient-specific navigational template for accurate prosthesis implantation in THRA by computer-aided technology and reverse engineering, and verifying its outcomes in cadavers. Consequently, individual treatment is achieved accurately and effectively.

## Methods

### Data acquisition

Seventeen specimens with the lower extremities (10 males and 7females aged from 45 to 65 years) were obtained from the Department of Anatomy at Kunming Medical University. All specimens were examined by fluoroscopy to exclude other hip diseases and deformities. A spiral 3D computed tomography scan (Light Speed VCT, General Electric, USA) was performed on each specimen with a 0.630 mm slice thickness and 0.35 mm in-plane resolution (100 mA tube current; 120 kV tube voltage; 15 s to 20 s scan time; 512 × 512 scan matrix). All scans were performed with the specimens in a supine position. All imaging data were stored in the DICOM (digital imaging and communications in medicine) format.

### Preoperative planning

**Fig. 1:**
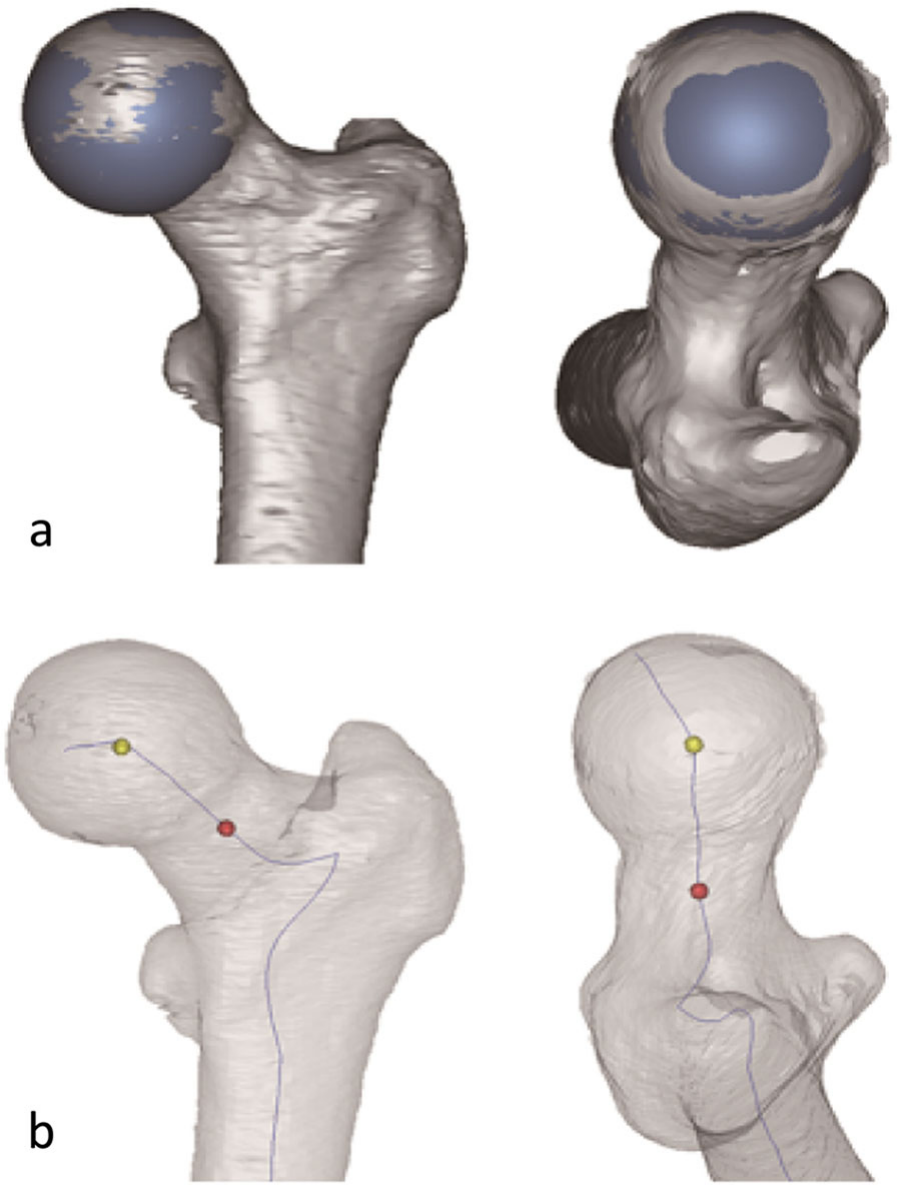
Calculating the anatomical central axis of femoral neck. **a** Calculating the femoral head center by fitting a suitable sphere (yellow point). **b** Calculating the the proximal femur centerline and the femoral neck center (red point)

Every single index hip joint with intact acetabulum and proximal femur was segmented and the 3D model was generated subsequently, using the MIMICS10.01 software (Materialise, Belgium). We fitted the femoral head into a standard sphere and obtained it’s center which was regarded as the femoral head center (Fig. 1a). Then, we marked the mid-point from the centerline of the femoral neck, which was selected as the femoral neck center (Fig. 1b). Two coordinates of the two centers were multi-calculated and the mean values were obtained. Then we obtained the central axis of the femoral neck.

**Fig. 2:**
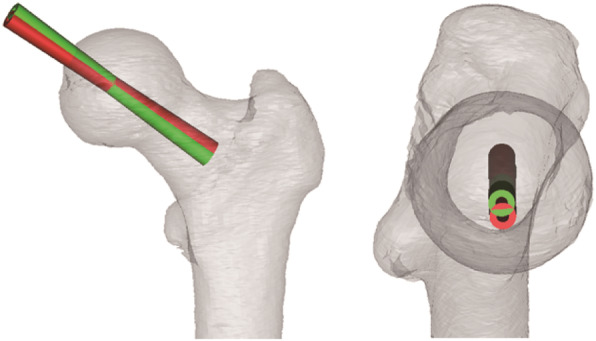
Virtual location of the drill guide. The initial position (red) was consistent with the central axis of the femoral neck. The final direction (green) was confirmed by a small valgus angle of 5°-10° around the femoral head center

A virtual drill guide with 4 mm diameter was placed according to the location of the femoral neck axis for placement simulation to obtain the optimal Kirschner wire trajectory on the 3D femoral model. The final direction of the drill guide was confirmed by a valgus angle of 5°- 10°, which served as the original point of the femoral head center on the coronal plane (Fig. 2). The optimal length of the drill guide was determined based on the measurement.

**Fig. 3:**
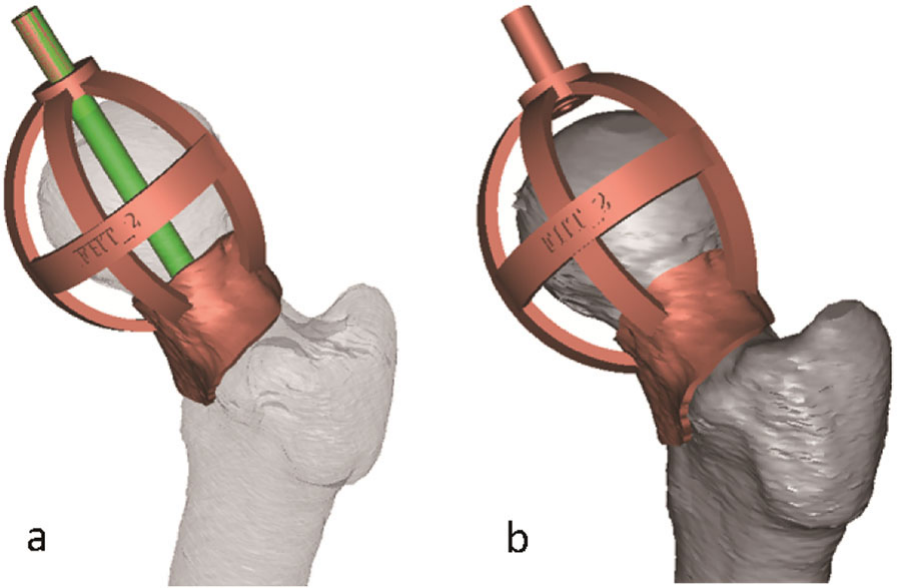
The design of navigational template. **a** The direction of the template was consistent in the drill guide. **b** The virtual surface of template matched the femoral neck surface perfectly

Second, the femur model was exported in stereolithograph (STL) format to a workstation running Geomagic Studio 12 software (Geomagic Inc., USA) to create the custom-made template surface according to the optimal drill guide trajectory. The surface was generated by extracting the inverse of the femoral neck surface, finally, a drill guide templated was established (Fig. 3). We did the re-check and ensured that the template did not involve in the adjacent fragments.

**Fig. 4:**
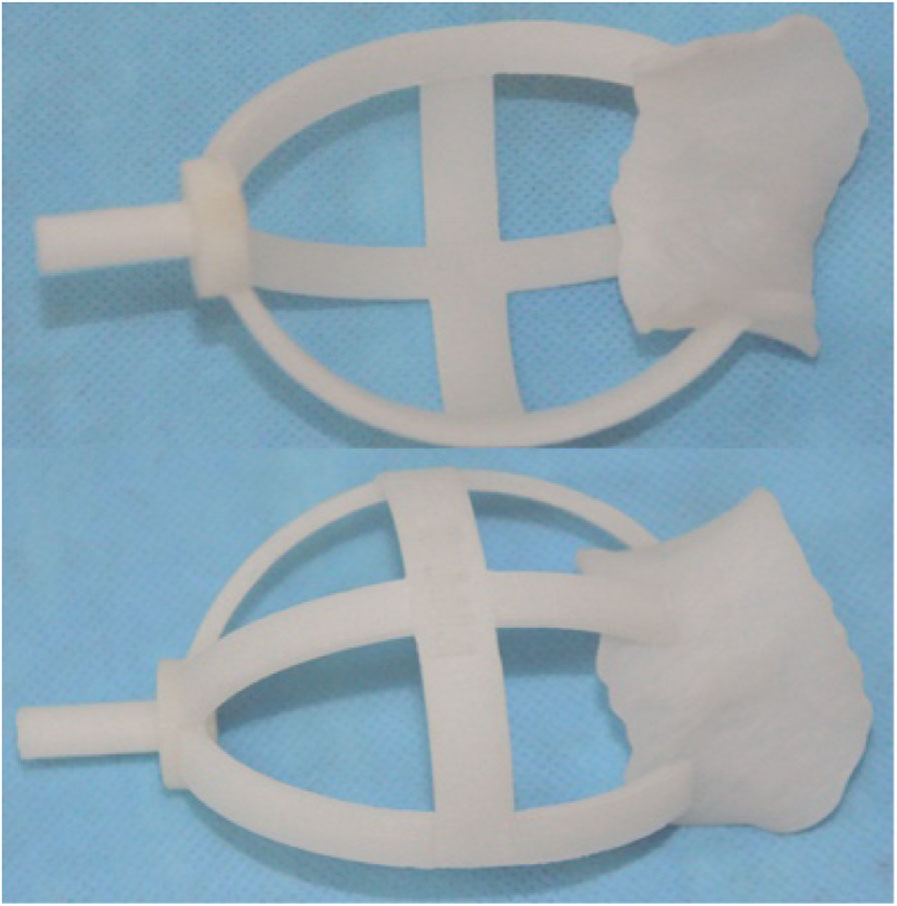
The actual model of the navigational template produced by rapid prototyping

Subsequently, a virtual custom-made navigational template that match the femoral neck surface was constructed with a drill guide as the navigational channel for the Kirschner wire. Inevitably, the Kirschner wire represented the expected position of the femoral head prosthesis. The bio-model of the novel navigational template was produced with medical-class acrylic resin (Somos 14,120, DSM Desotech Inc., USA) by stereolithography, which is a RP technique (Bing Chuang Company, China) (Fig. 4). System parameters were set as follows: 0.1 mm processing layer thickness, 450 mm/s processing speed, and 45 °C ± 2 °C temperature.

### Surgical methods

**Fig. 5:**
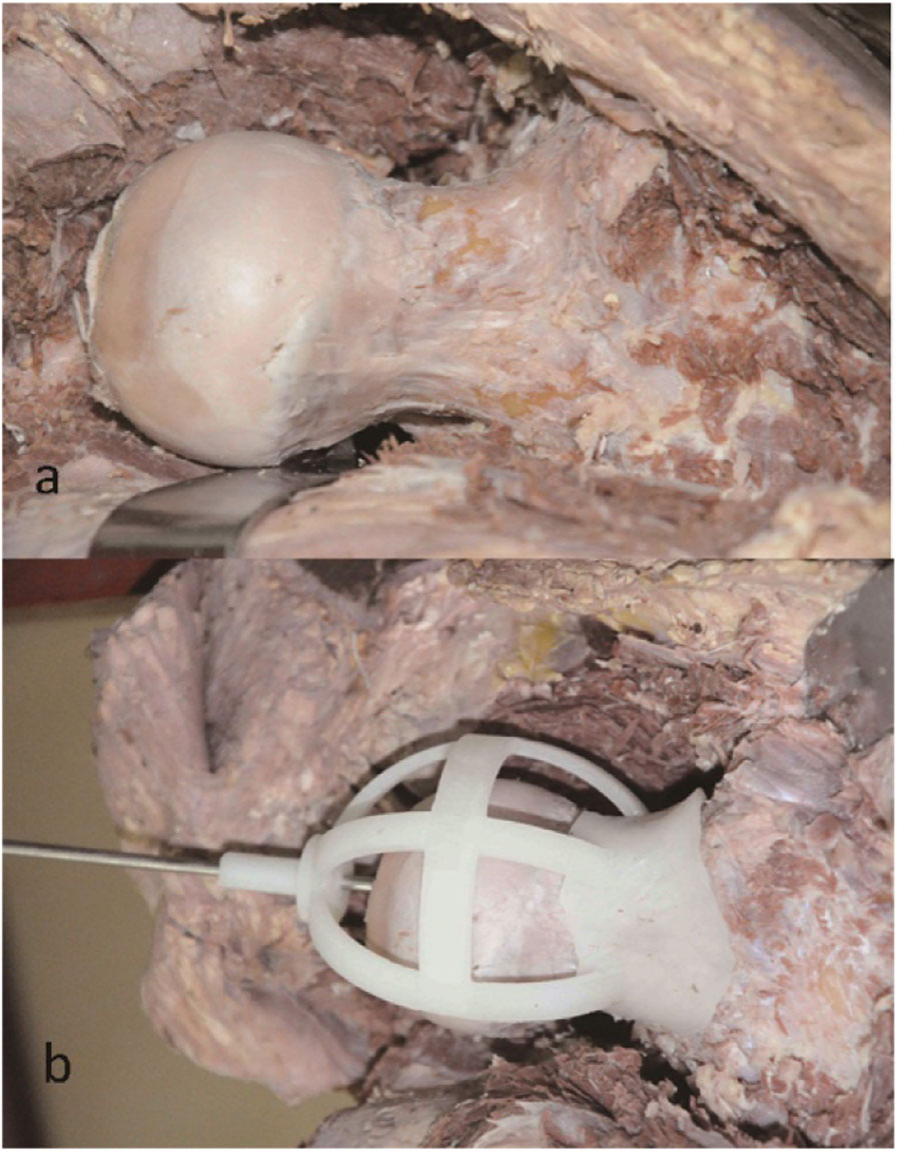
Cadaveric experiment. a The exposure and dislocation of the femoral head. **b** The navigational template fitted the femoral neck perfectly and a Kirschner wire was nailed in for fixation

All the cadaver experiments were performed in a laboratory by the senior author (LJL, LS) in accordance with the following procedures. Bodies were placed in a lateral decubitus position on a table and exposed as necessary prior to hip joint dislocation (Fig. 5a). The navigational template was then matched to the femoral neck surface perfectly. Thereafter, a high-speed drill was used to drill a trajectory of the Kirschner wire along the guidance sleeve (Fig. 5b). Finally, a Kirschner wire was inserted into the femoral head as the expected channel of prosthetic implantation.

### Statistical analysis

The positions of the Kirschner wires after the surgeries were evaluated by x-ray and CT scan. All data were measured by AutoCAD2010 (Auto Desk, USA) and presented in the form of mean ± standard deviation (SD). Statistics software Prism 6.0 (GraphPad Software, US) was employed to analyze the data. Measurement data subject to or approximately subject to normal distribution were expressed as mean ± SD, and paired-T-test was used to compare the preoperative Neck-Shaft Angle (NSA) and the postoperative Stem-Shaft Angle (SSA).

Measurements have been recorded as follow: (1) Stem-shaft angle (SSA):the angle between the axial line of the femur shaft and extension line of the Kirschner wire; (2) lateral offset: the acute angle between the central axis of the femoral neck and the implanted Kirschner wire in the coronal plane; It is used to evaluate whether the guide wire has varus or valgus. (3) horizontal offset: the acute angle between the central axis of the femoral neck and implanted Kirschner wire in the transverse plane. It is used to evaluate whether the guide wire has antero-posterior deviation.

## Results

During the operation, we easily achieved the reliable matching between the template and the femoral neck. No significant free motion existed when the template was placed in. Thus, the template can be used as an in- situ drill guide for position fixation. Visual inspections showed that the actual surfaces match perfectly to the template of the virtual surface without any further restrictions.

A total of 17 cases were instrumented with Kirschner wires by using the novel navigational templates. In the coronary plane there had no significant valgus or varus angle occurred. The Kirschner wires passed through the center of the femoral head (16 cases). Compared to the femoral neck axis, 15 cases were with relative valgus ranging from 6.2° to 12.3°, 1 case was paralleled, 1 cases was varus. In the transverse plane there had no obvious horizontal deviation had occurred. The Kirschner wires passed through the center of the femoral head (17 cases). Compared to the femoral neck axis, 15 cases were approximately parallel, and there was 1 case of forward and backward deviation had occurred respectively.

**Fig. 6:**
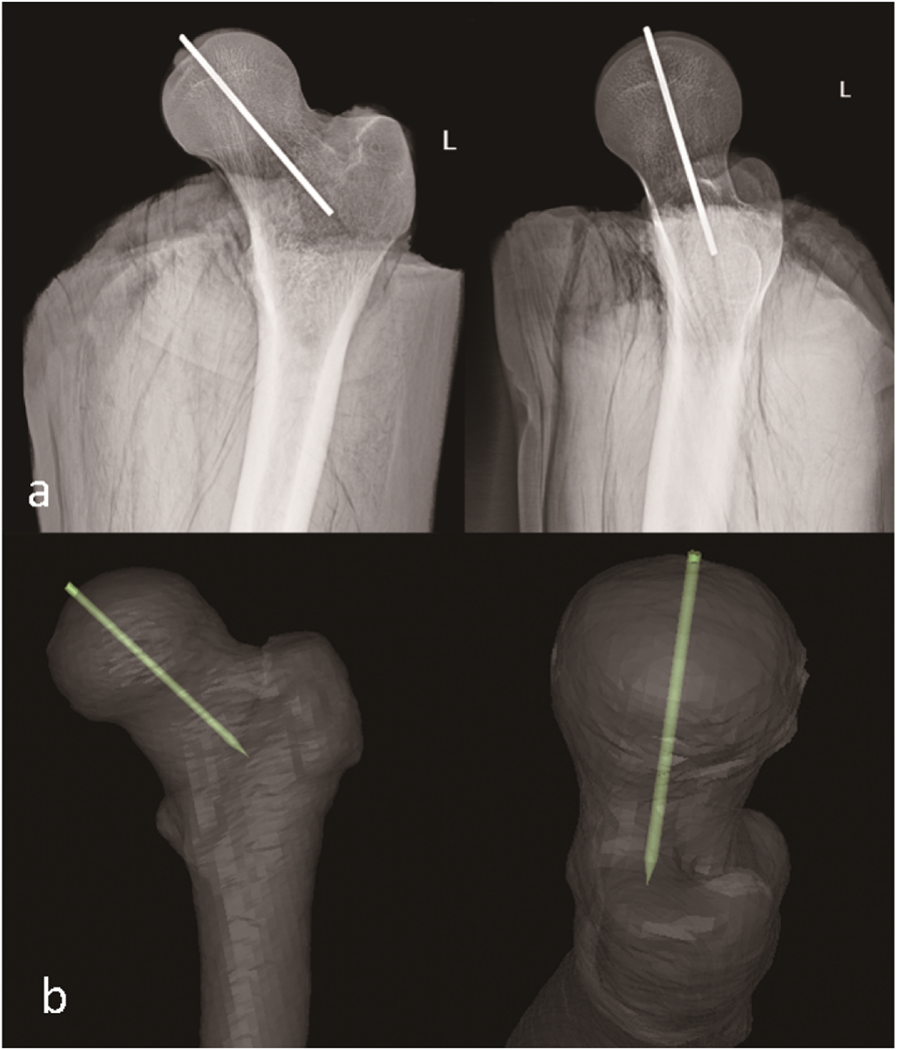
Postoperative x-rays and CT reconstruction show the perfect and accurate position of Kirschner wire. **a** Postoperative x-rays show the anteroposterior and lateral position of the Kirschner wire. **b** 3D reconstruction of postoperative CT scan shows the optimal postion of the Kirschner wire

**Fig. 7:**
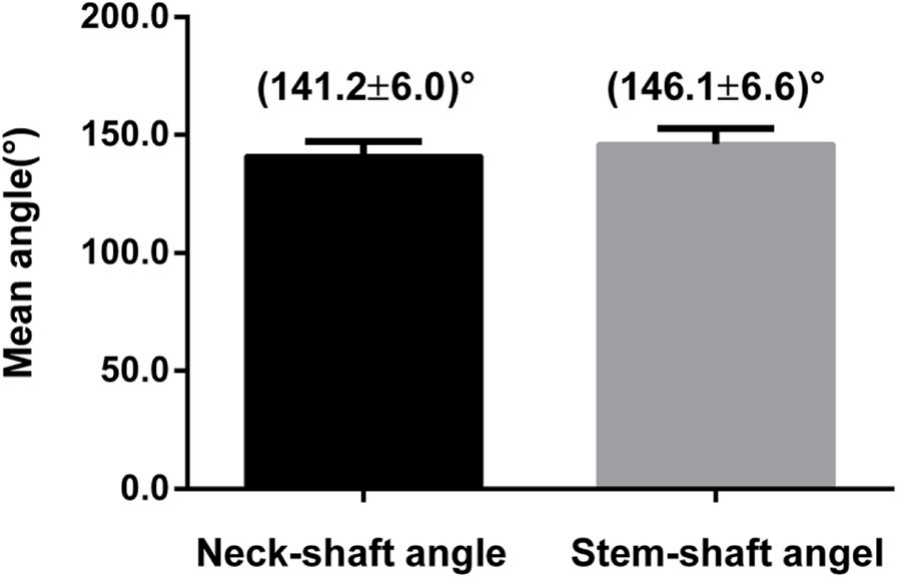
The NSA preoperative and SSA postoperative. Data are expressed as mean ± SD, and there is no significant difference (*P* > 0.05)

**Fig. 8:**
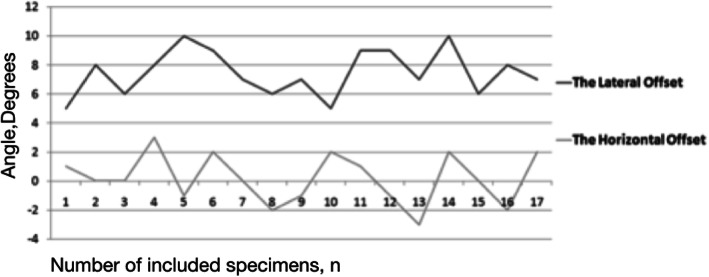
Folded diagram of the lateral offset and the horizontal offset. The horizontal coordinates describes the number of included specimens, and the vertical coordinates describes the degree of lateral offset angle (black) and horizontal offset angle (gray) postoperative. As we can see from the diagram, the lateral offset shows expected requirement of relative valgus angle was achieved, the horizontal offset shows no obvious antero-posterior deviation had occured

As the results indicated, the average valgus angles of the Kirschner wires (8.7° ± 1.7°) were obtained by comparing the preoperative Neck-Shaft Angle (NSA)(141.2° ± 6.0°) with the postoperative Stem-Shaft Angle (SSA) (146.1° ± 6.6°) of all samples (Figs. 6, 7 and 8). And there is no significant difference (*P* > 0.05).

## Discussion

THRA is an alternative method to THA for younger and more active patients with hip diseases [15, 16]. It enables the preservation of an intact femoral neck, closely mimics the normal anatomy of proximal femur, optimizes stress transfer, offers inherent stability and optimal range of movement [17, 18]. The main failure factors include femoral neck fracture and the aseptic loosening of the femoral prosthesis [19]. The femoral neck notch and varus position of the prosthesis caused by improper operation increases the risk of failure [20–22]. Implant position significantly influences implant survival, patient outcomes, and prosthesis life span [23]. Careful and individualized preoperative planning ensures proper implant position because of the different anatomies of the hip joint [13].

In conventional THAR, the surgeon usually achieves the implant channel for the prosthesis manually mostly based on their experience [24]. Thus, accumulated experience is important before a surgeon can apply THAR. Furthermore, the alteration of the position can cause negative effects on the accuracy of the prosthesis. The offset of the femoral neck central axis always occurs when a traditional localizer is used, thus increasing the risk of fracture. Computer-aided technology often requires additional components, such as displays, sensors, and robot systems, for the intraoperative registration of bone structures. Not all hospitals can meet the costs of these devices [13], and no concise and effective method can ensure the accurate implantation of hip resurfacing systems in contemporary.

We utilized computer-aided technology, RP, and RE to design a new custom-made navigational template to eliminate the need for expensive and complex devices, facilitate accurate placement, and achieve satisfactory results. The spherical center of the fitting sphere approaches significance to the anatomical center of the femoral head by using the contour lines of the femoral head cortex. Despite of the irregular geometry of the femoral neck, the centerline inevitably passes the center of each tangent plane and is perpendicular to the major axis of the femoral neck and the proximal femur. Thus, we chose the point by fitting the sphere center of the femoral head and the mid-point on the centerline of the femoral neck to determine the axis of the prosthesis. The template also has an unlimited oncoming meaning between the anatomical axis of the femoral neck and ligature of the two points. More accurate results can be achieved by repeated calculation and fitting. In general, the pathological femoral head can be abnormal due to the osteophytes, and the articular cartilage can have a negative effect on the matching accuracy. Therefore, we chose the femoral neck region as the inverse of the template surface, and optimal matching between the template and the femoral neck would be achieved, considering the obvious anatomical mark, easy exposure, and less cartilage,.

It is important for the valgus orientation of the femoral component relative to the native femoral neck in THRA [25, 26]. When the valgus angle is set to approximately 140° or is anatomically anteverted, lateral neck and head interfacial stresses will be minimized. Excessive varus–valgus angles increase the rate of femoral neck fractures and prosthetic loosening [27]. Excessive antero-posterior deviation can also change the optimal mechanic axis. Thus, the final direction of the drill guide was confirmed by an appropriate valgus angle to mimic closely the pre-disease physiological status of the proximal femur. All the performances were based on preoperative CT scan, and we obtained the individualized preoperative implant planning. Postoperative evaluation reveals that the Kirschner wires pass through the center of the femoral head, have a relative expected valgus angle to the femoral neck central axis and have no obvious antero-posterior deviation. Thus, the Kirschner wires can be positioned as expected.

RP is a digital modeling technique based on the principle of separation and involves the accumulation of materials to create a prototype. RP is controlled by a computer and is based on computer-aided design models or imaging data from CT or magnetic resonance imaging (MRI). The manufacturing technique is precise to the point of 0.1 mm or less. Thus, the manufactured inverse surface of the template is the accurate replica of biological objects with similar morphologies.

In summary, our navigational template system serves as a novel alternative for the placement of hip resurfacing prosthesis. Our system is easy to implement, appropriate for surgeons without special training, eliminates time-consuming procedures in the operating room. The cost can be significantly reduced, compared with the computer assisted surgery instruments. The time consumed from the design to the production of one template is approximately 3 h. The addition of software expenses increases the price of the template. However, the software can be used in other research and clinical work, thus reducing the cost of the template in the long term. Thus, the template is a simple and low-cost solution for the accurate, safe, and rapid implementation of elective surgery on bone structures.

The limitations in this study are derived from various factors. First, the articular cartilage is not imaged by CT, thus causing a negative impact on matching accuracy. We can delineate the thickness of the articular cartilage via MRI in further research. Second, the design procedure of the template, including reconstruction and fitting, requires manual or automatic segmentation, which causes errors. Third, the bio-model of the template can deviate from the computer 3D model. This kind of error is related to the RP equipment and material. We controlled the precision within 0.1 mm in the rapid formation. Finally, the template should adhere tightly to the osseous marker during surgery because any movement between the bones affects the accuracy. This type of error represents the largest source of error in the entire process and should be controlled precisely.

Our study is a pilot trial with a limited number of cases. The application of this study only remains in basic research. Further studies are necessary to determine the appropriate values for clinical applications.

## Conclusions

The novel custom-made navigational template of femoral head can effectively assist surgeons for accurately implanting the femoral head components to the desired position in THRA.

## Data Availability

The datasets generated and/or analysed during the current study are not publicly available due to the data is confidential patient data but are available from the corresponding author on reasonable request.

## Abbreviations

THRA: Total hip resurfacing arthroplasty
CT: Computed tomography
RP: Rapid prototyping
MM-RA: Metal-on-metal hip resurfacing arthroplasty
RE: Reverse engineering
DICOM: Digital imaging and communications in medicine
STL: Stereolithograph
SSA: Stem-shaft angle
NSA: Neck-shaft angle
MRI: Magnetic resonance imaging

## References

[1] Crowninshield RD, Rosenberg AG, Sporer SM (2006). Changing demographics of patients with total joint replacement. Clin Orthop Relat Res.

[2] Thillemann TM, Pedersen AB, Mehnert F (2010). Postoperative use of bisphosphonates and risk of revision after primary total hip arthroplasty: a nationwide population-based study. Bone..

[3] Flecher X, Parratte S, Brassart N (2008). Evaluation of the hip Center in Total hip Arthroplasty for old developmental dysplasia. J Arthroplast.

[4] Clohisy JC, Dobson WA, Robison JF (2011). Radiographic structural abnormalities associated with premature, natural hip-joint failure. J Bone Joint Surg Am.

[5] Girard J (2016). Hip resurfacing: international perspectives: review article. HSS J.

[6] Tao R, Liu F, Liu YK, et al. A prospective comparative study of hip resurfacing arthroplasty and large-diameter head metal-on-metal total hip arthroplasty in younger patients-a minimum of five year follow-up. Int Orthop. 2018;42(10):2323–7. 10.1007/s00264-018-3819-9 29455347

[7] Amstutz HC, Campbell PA, Duff MJL (2004). Fractures of the neck of thefemur after surface arthroplasty of the hip. J Bone Joint Surg Am.

[8] Amstutz HC, Campbell PA, Duff MJ (2006). Incidence and prevention of femoral neck fractures after hybrid metal-on-metal hip resurfacing [J]. J Bone Joint Surg Br..

[9] Kruger S, Zambelli PY, Leyvraz PF (2009). Computer assisted placement technique in hip resurfacing arthroplasty: improvement in accuracy?. Int Orthop.

[10] Lin HH, Lonic D, Lo LJ (2018). 3D printing in orthognathic surgery - a literature review. Taiwan Yi Zhi.

[11] Zhang G, Yu Z, Chen X (2018). Accurate placement of cervical pedicle screws using 3D-printed navigational templates: an improved technique with continuous image registration. Orthopade..

[12] Sun WY, Zhang L, Wang L (2019). Three-dimensionally printed template for percutaneous localization of multiple lung nodules. Ann Thorac Surg.

[13] Zhang YZ, Lu S, Xu YQ (2011). Design and primary application of computer-assisted, patient-SpecificNavigational templates in metal-on-metal hip resurfacing Arthroplasty. J Arthroplast.

[14] Raaijmaakers M, Gelaude F, Smedt KD, et al. A custom-made guide-wire positioning device for hip surface replacement Arthroplasty: description and first results. BMC Musculoskelet Disord. 2010;11:161–8. 10.1186/1471-2474-11-161 PMC2913994 20630093

[15] Olsen M, Gamble P, Chiu M (2010). Assessment of accuracy and reliability in preoperative Templating for hip resurfacing Arthroplasty. J Arthroplast.

[16] Buergi ML, Walter WL (2007). Hip resurfacing Arthroplasty. The Australian Experience. J Arthroplasty.

[17] Back DL, Dalziel Y, Young D (2005). Early results of primary Binningham hip resurfac-ings. An independent prospective study of the first 230 hips. J Bone Joint surg Br.

[18] Little JP, Taddei F, Viceconti M (2007). Changes in femur stress after hip resurfacing arthroplasty: response to physiological loads. Clin Biomech.

[19] Bos PK, Biezen FCV, Weinans H (2011). Femoral Component Neck Fracture After Failed Hip Resurfacing Arthroplasty. J Arthroplasty.

[20] Shimmin AJ, Back D (2005). Femoral neck fractures following Birmingham hip resurfacing: a national review of 50 cases. J Bone Joint Surg Br.

[21] Marker DR, Seyler TM, Jinnah RH (2007). Emoralneck fractures after metal-on-metal total hip resurfacing: a prospective cohort study. J Arthroplast.

[22] Anglin C, Masri BA, Tonetti J (2007). Hip resurfacing femoral neck fracture influenced by valgus placement. Clin Orthop Relat Res.

[23] Hurst JM, Millett PJ (2010). A simple and reliable technique for placing the femoral neck guide pin in hip resurfacing Arthroplasty. J Arthroplast.

[24] Daniel J, Pynsent PB, Mcminn DJW (2004). Metal-on-metal resurfacing of the hip in patients under the age of 55 years with osteoarthritis. J Bone Joint Surgery Br.

[25] Shimmin AJ, Bare J, Back DL (2005). Complications associated with hip resurfacing Arthroplasty. Orthop Clin North Am.

[26] Long JP, Bartel DL (2006). Surgical variables affect the mechanics of a hip resurfacing system. Clin Orthop Relat Res.

[27] Ong KL, Day JS, Kurtz SM (2009). Role of surgical position on Interface stress and initial bone remodeling stimulus around hip resurfacing Arthroplasty. J Arthroplast.

